# Efficacy and safety of Chinese herbal medicine in treating postcholecystectomy diarrhea: A systematic review and meta-analysis

**DOI:** 10.1097/MD.0000000000038046

**Published:** 2024-05-03

**Authors:** Yan Sun, Yong Zhang, Zheng Wang, Quanda Liu, Juefei Mo

**Affiliations:** a Guang’an Men Hospital, China Academy of Chinese Medical Sciences, Xicheng District, Beijing, P.R. China.

**Keywords:** Chinese herbal medicine, conservative treatment, postcholecystectomy diarrhea, systematic review

## Abstract

**Background:**

Postcholecystectomy diarrhea (PCD) is among the most distressing and well-known clinical complications of cholecystectomy. Despite various available treatment options, clinical outcomes are greatly limited by unclear pathophysiological mechanisms. Chinese herbal medicine (CHM) is widely used as a complementary and alternative therapy for the treatment of functional diarrhea. Thus, we conducted a meta-analysis of randomized controlled trials (RCTs) to evaluate the efficacy and safety of CHM for the treatment of PCD.

**Methods:**

Electronic database searches were conducted using the Cochrane Library, PubMed, Web of Science, Embase, Wanfang Data, China National Knowledge Infrastructure, and the Chinese Scientific Journal Database. All RCTs on CHMs for managing patients with PCD were included. The meta-analysis was performed using RevMan 5.4 software.

**Results:**

The present meta-analysis included 14 RCTs published between 2009 and 2021 in China. The primary findings indicated that CHM had a higher total efficacy and cure rate as a monotherapy for PCD (*P* < .00001). Two trials reported the scores of the main symptoms with statistically significant differences in stool nature (*P* < .00001), defecation frequency (*P* = .002), and abdominal pain and bloating (*P* < .00001). In addition, CHM reduced CD_3_^+^ and CD_4_^+^ levels more effectively in terms of T lymphocyte subset determination (*P* < .00001). The main symptoms of PCD in traditional Chinese medicine (TCM) are splenic deficiency and liver stagnation. All treatments were used to strengthen the spleen and (or) soothing the liver.

**Conclusion:**

CHM had a favorable effect on PCD. No adverse events were observed. Larger, high-quality RCTs are warranted to draw definitive conclusions and standardize treatment protocols.

## 1. Introduction

Cholecystectomy remains the gold-standard method of therapy because of the increasing incidence of benign gallbladder diseases, mainly cholecystitis and gallstones.^[1]^ Moreover, the laparoscopic approach has been widely used as the standard technique because it has significantly lower morbidity and mortality rates than open surgery.^[2,3]^ Nevertheless, postcholecystectomy syndrome (PCS) may occur weeks or months after cholecystectomy and manifests with clinical symptoms such as diarrhea, abdominal pain, and bloating. In particular, postcholecystectomy diarrhea (PCD) is a relatively well-known clinical complication^[4,5]^ characterized by a prolonged course, recurrence, and persistence, which significantly affects the quality of life of patients and even causes a portion of the population to develop disability.^[6]^ PCD is highly prevalent and protracted. A previous review reported an average prevalence of 13.3% (2.1%–57.2%), although with the limitation of various follow-up times.^[4]^ The 2020 Canadian Chronic Diarrhea Guidelines highlight cholecystectomy as a risk factor for chronic diarrhea.^[7]^ In addition, Ribas et al^[8]^ found that >50% of patients had abnormal bowel habits after cholecystectomy, of which 23% had unrelieved symptoms after 6 months.

The pathophysiological mechanisms underlying PCD are not well understood. Despite different treatment options available, clinical outcomes are unsatisfactory. Due to their expanding role in the primary management of gastrointestinal diseases, dietary interventions are recommended.^[9]^ However, a recent study showed that a low-lipid diet did not improve the symptoms in patients with PCD.^[8]^ In addition, owing to the lack of sufficient clinical data and negative publicity of media reports, there is no conclusive evidence of the true clinical effect of dietary interventions.^[10]^ In addition, nearly a quarter of patients with PCD take different types of bile-binding drugs, of which colestyramine is the most frequently used. However, a systematic review including a small number of studies showed that the short- and long-term responses to colestyramine in the postcholecystectomy group were 47.63% and 36.2%, respectively. Notably, only 47.52% of patients with a clear diagnosis of severe bile acid diarrhea had a higher short-term response,^[11]^ and the long-term response was largely limited by poor tolerance.^[12]^ Fecal microbiome transplantation is a promising therapy for irritable bowel syndrome (IBS) that involves rebuilding the intestinal microbiota, restoring intestinal function, and repairing the intestinal mucosa.^[13]^ Nevertheless, currently, randomized controlled trials (RCTs) showed conflicting results.^[14,15]^ In addition, probiotics are not recommended because of higher-quality evidence and differences in the composition of probiotics.^[10]^

Traditional Chinese medicine (TCM) has long used a combination of herbal remedies and multiple targets based on symptom patterns.^[16,17]^ The variety of Chinese herbs is rich, with the advantages of being well tolerated, simple to produce, and economical. Indeed, Chinese herbal medicine (CHM) was preliminarily acknowledged for functional diarrhea in the clinic. Considering the disabling nature and the lack of effective treatments for PCD, CHM has been widely used as a complementary and alternative therapy. However, systematic analyses of CHM in PCD are lacking. Thus, we conducted a meta-analysis of RCTs to evaluate the efficacy and safety of CHM in treating PCD and attempted to provide a protocol for clinical practice.

## 2. Materials and methods

### 2.1. Criteria for including studies

All RCTs on CHMs for managing patients with PCD were included. Specifically, studies on CHMs versus Western medicines or blank controls were included. The outcomes included the total effective rate, cure rate, T lymphocyte subset determination, and scores for the main symptoms.

### 2.2. Criteria for excluding studies

Studies on other main treatments were excluded. Non-RCTs, non-AIS, clinical experience, trials with fewer than 10 patients, cross-sectional studies, case reports, comments, and reviews were excluded from the analysis.

### 2.3. Database searches

Electronic database searches were conducted from database inception to October 2022, including Cochrane Library, PubMed, Web of Science, Embase, Wanfang Data, China National Knowledge Infrastructure, and Chinese Scientific Journal Database. The combinations of MeSH Terms and relevant keywords were as follows: “Cholecystectomy” (MeSH Terms) AND “Diarrhea” (MeSH Terms) OR “Postcholecystectomy Syndrome” (MeSH Terms). To comprehensively include the literature, we manually screened the target literature regarding CHM for managing patients with PCD. Additionally, a search strategy was determined for each database. The language was restricted to English or Chinese, with no limitations on the subheadings. We searched the reference lists of the identified papers to explore other studies, and trials not covered in the aforementioned databases were additionally searched once they were identified. Duplicate studies were excluded after reviewing the abstracts and full texts. This study mainly referred to the 12 reporting guidelines provided by the Preferred Reporting Items for Systematic Reviews and Meta-Analyses for the meta-analysis of intervention trials.^[18]^

### 2.4. Data collection and analysis

Data processing was independently managed by 2 authors using Endnote X8 software, and disagreements were resolved by a third author. The information for each eligible study included: descriptive statistics such as author information, publication year and country, data sources, and sample sizes; intervention characteristics such as detailed CHM and treatment course; type of clinical study design and methods of randomization and blinding; and information on outcomes such as outcomes of interest, follow-up duration, and adverse events. The total effective rate was calculated as the ratio of the number of patients with positive outcomes to the total number of patients, whereas the cure rate was the ratio of the number of cured patients to the total number of individuals. If necessary, we contacted the authors of the included studies for additional original data. According to the guiding principles of clinical research on new TCM: Trial,^[19]^ clinical symptom scores, including stool properties, defecation frequency, and abdominal pain and bloating, were observed in the 2 groups. A score of 0 was classified as asymptomatic, 1 as mild, 2 as moderate, and 3 as severe. Lower scores indicate less severe clinical symptoms.

### 2.5. Statistical analysis

The meta-analysis was performed using RevMan 5.4 software. Statistical heterogeneity was evaluated using the chi-square and *I^2^* tests. An *I^2^* value of <25% indicated low heterogeneity and that <50% indicated moderate heterogeneity. A fixed-effects model was used. Otherwise, an *I^2^* value >50% indicated significant heterogeneity, and a random-effects model was adopted. Standardized mean differences (SMDs) of 95% confidence intervals (CIs) were used for different measurement methods. If significant heterogeneity was observed, a sensitivity analysis was performed.

## 3. Results

### 3.1. Literature search

**Figure 1.**
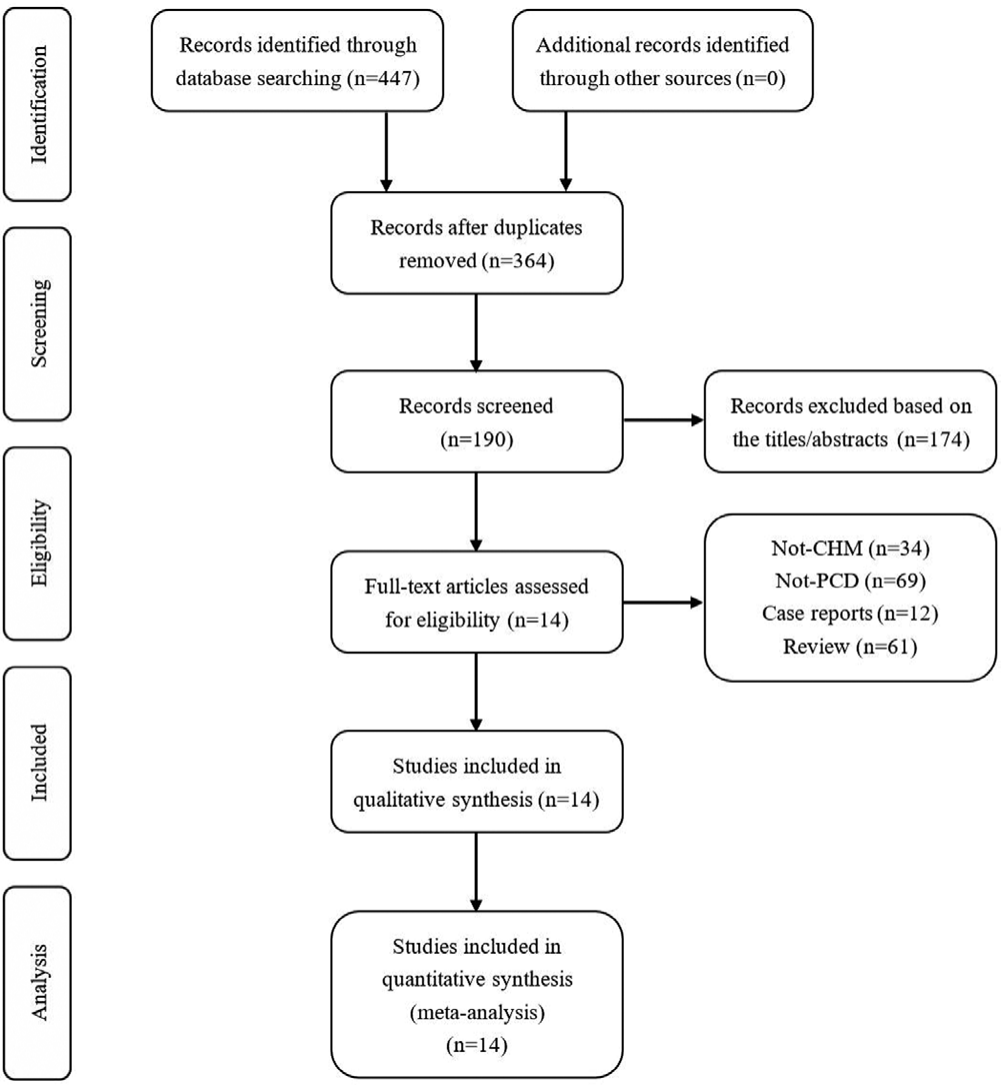
Flow diagram of the selection process for the studies included in the meta-analysis.

First, 447 studies were confirmed using PubMed (n = 14), the Cochrane Library (n = 32), China National Knowledge Infrastructure (n = 222), Chinese Scientific Journal Database (n = 51), Embase (n = 34), and Wanfang Data (n = 94). Subsequently, we independently reviewed the abstracts and titles and removed duplicates, resulting in 190 studies. On the basis of the inclusion criteria, non-RCTs, reviews, opinions, and records with inappropriate intervention approaches were excluded. After reading the full texts, 176 studies were excluded for the following reasons: non-RCTs (n = 73), intervention measures other than traditional Chinese exercises (n = 34), and patients with other serious diseases (n = 69). Ultimately, 14 RCTs^[20–33]^ were included in the analysis. No additional studies were included in the reference list (Fig. 1). The characteristics of the included studies are listed in Table 1.

**Table 1:** Characteristics of all the trials included in the meta-analysis.

Study ID	Country	Sample size (M/F)		Age (yr old)		Course		Intervention		Treatment time (post-op)	Treatment duration
		I	C	I	C	I	C	I	C		
Miao 2011^[20]^	China	7/15	8/14	51 (28–81)	49 (26–78)	>3 mo		TXYF + SLBZP, 200 mL twice daily	BLC, 50 mg thrice daily	>3 mo	1 mo
Hu 2015^[21]^	China	21	15	43.2		4.2 (0.5–10) yr		BZYQD, thrice daily	BLC + CDEC	0.5–10 yr	6 wk
Zhu 2009^[22]^	China	15/41	19/37	52.1	50.7	NR		JPYQD, twice daily	blank	6 d	2 wk
Liu 2014^[23]^	China	39	39	38.2 ± 9.8		3–12 mo		LZD + TXYF, 200 mL twice daily	MP, 300 mg thrice daily	3–12 mo	2 mo
Lin 2013^[24]^	China	10	10	45.2 ± 8.7		NR		SLBZP, 500 mL once daily	Loperamide, 500 mg per daily	<5 d	2 mo
Wang 2014^[25]^	China	16/24	18/22	47.4 ± 9.7	49.1 ± 10.3	14.6 ± 3.7 mo	13.4 ± 3.5 mo	XYPWD	LCBLT, 100 mg thrice daily + MP, 300 mg thrice daily	3–12 mo	2 wk
Zhuang 2016^[26]^	China	21/24	20/25	46.9 ± 8.8	47.5 ± 7.6	3.3 ± 0.8 yr	3.5 ± 0.6 yr	JPZSD, 250 mL twice daily	LCBLT, 200 mg thrice daily + MP, 300 mg thrice daily	NR	3 mo
Li 2016^[27]^	China	7/9	6/10	48.5 (45–55)	48.3 (44–55)	3–12 mo		SLBZP, twice daily	MP, 300 mg thrice daily	3–12 mo	2 mo
Zhang 2019^[28]^	China	17/13	18/12	51.6 ± 2.1	52.2 ± 2.4	>1 mo		TXYF, twice daily	CBT, 700 mg thrice daily + PBT, 50 mg thrice daily	>1 mo	NR
Yao 2017^[29]^	China	15/21	17/19	47.5 ± 7.6	46.9 ± 8.5	3.6 ± 0.6 yr	3.3 ± 0. 8 yr	TXYF + SSP + GGQLD, 200 mL twice daily	BTVCDI, 420 mg thrice daily + MP, 300 mg thrice daily	NR	1 mo
Zou 2019^[30]^	China	21/19	22/18	45.9 ± 5.8	47.2 ± 6.1	18.4 ± 6.7 mo	17.3 ± 5.2 mo	TXYF + SJZD, twice daily	BTVCDI, 200 mg thrice daily	>12 mo	2 mo
Wu 2018^[31]^	China	27/28	28/27	48.5 ± 8.2	50.5 ± 12.5	NR		SLBZP, twice daily	MP, 300 mg thrice daily	2–5 d	NR
Cheng 2018^[32]^	China	21/19	18/22	38.46 ± 5.89	39.48 ± 7.54	4.39 ± 4.09 yr	4.47 ± 4.14 yr	XXD, 200 mL twice daily	BTVCDI, 420 mg twice daily	NR	3 mo
Xiang 2021^[33]^	China	15/15	14/16	52.69 ± 2.31	52.24 ± 2.26	4.92 ± 0.68 yr	5.15 ± 0.82 yr	TXYF + SJZD, twice daily	BTVCDI, 840 mg thrice daily	2–11 mo	2 mo

BLC = Bacillus Licheniformis Capsule, BTVCDI = Bifid Triple Viable Capsules Dissolving at Intestines, BZYQD = Buzhong Yiqi Decoction, CBT = Clostridium Butyricum Tablets, CDEC = Compound Digestive Enzyme Capsules, GGQLD = Gegen Qinlian Decoction, JPYQD = Jianpi Yiqi Decoction, JPZSD = Jianpi Zaoshi Decoction, LCBLT = Live Combined Bifidobacterium and Lactobacillus Tablets, LZD = Lizhong Decoction, MP = Montmorillonite Powder, NR = not reported, PBT = Pinaverium Bromide Tablets, SJZD = Sijunzi Decoction, SLBZP = Shenling Baizhu Powder, SSP = Sishen Pill, TXYF = Tongxie Yaofang, XXD = Xiexie Decoction, XYPWD = Xiaoyao Pingwei Decoction.

### 3.2. Study characteristics

The present meta-analysis included 14 RCTs published between 2009 and 2021 in China. All studies focused on the efficacy of independent CHM in the treatment of PCD compared to the control group. The CHM used in the selected studies had great heterogeneity, but all were consistent with the syndrome type of spleen deficiency and the liver stagnation pattern. Patients were treated for 2 weeks, 1 month, 6 weeks, 2 months, and 3 months in 2, 2, 1, 5, and 2 studies, respectively. In all studies, the types and frequencies of CHM were not completely consistent, and the Western medicine groups were different. Specifically, patients were treated with Tongxie Yaofang in 5 studies, Shenling Baizhu Powder in 4 studies, Sijunzi Decoction in 2 studies, and all the others including Buzhong Yiqi Decoction, Jianpi Yiqi Decoction, Xiaoyao Pingwei Decoction, Jianpi Zaoshi Decoction, Sishen Pill, Gegen Qinlian Decoction, Lizhong Decoction, and Xiexie Decoction appeared only in 1 study. The drugs used in the control groups were Bacillus licheniformis capsules, compound digestive enzyme capsules, montmorillonite powder, live combined bifidobacteria and lactobacillus tablets, Clostridium butyricum tablets, pinaverium bromide tablets, and bifid triple-viable capsules dissolved in the intestines. Treatment initiation ranged from 2 days to 12 months. The most commonly used CHM consists of Tongxie Yaofang and Shenling Baizhu Powder, which are used for treating liver stagnation and spleen deficiency patterns of syndrome, respectively. All trials reported a total effective rate, 13 trials reported a cure rate, 3 reported T lymphocyte subset determination, and 2 reported the scores of the main symptoms. All included studies were based on the criteria of diagnosis and therapeutic effect of diseases and syndromes in TCM to evaluate the efficacy of drugs, especially CHM, for PCD.

### 3.3. Risk of bias

**Figure 2.**
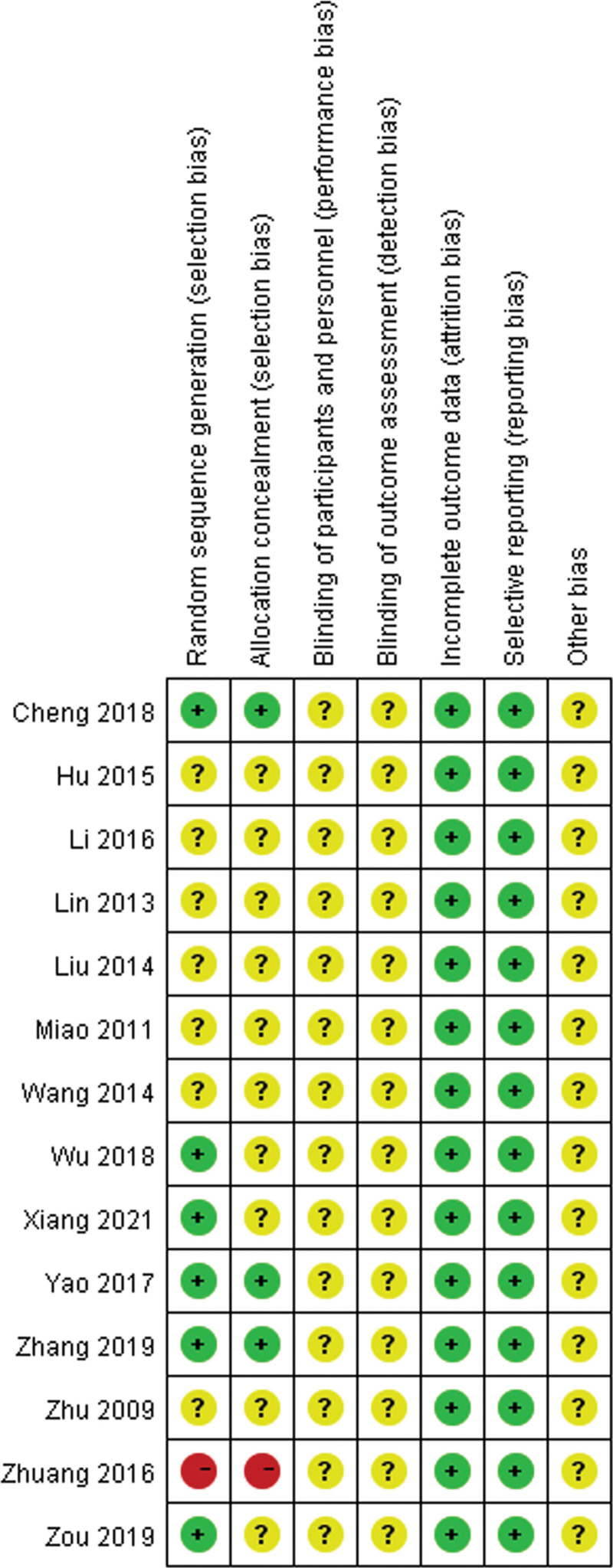
Risk of bias summary: +, low risk of bias; −, high risk of bias; ?, bias unclear.

Of the 14 included studies, all but one were considered to have a low risk of bias. Random sequence generation was reported in 6 studies and allocation concealment was reported in 3 studies. Only 1 study had a high risk of bias in random sequence generation and allocation concealment. As shown in Figure 2, blinding of participants and personnel, blinding of the outcome assessment, incomplete outcome data, selective reporting, and other biases were not observed in any of the 14 studies.

### 3.4. Outcome measures

#### 3.4.1. Total effective rate

**Figure 3.**
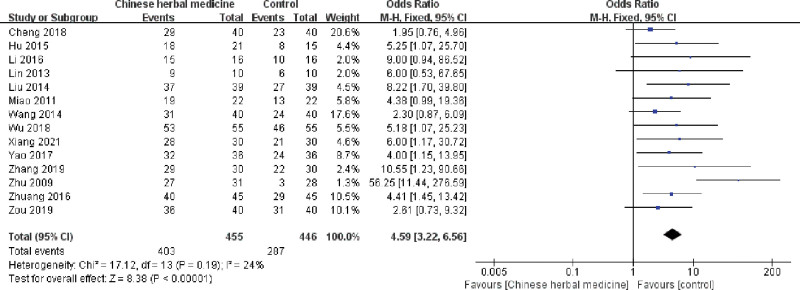
Forest plot showing the effect of Chinese herbal medicine vs controls on the total effective rate in the treatment of postcholecystectomy diarrhea (CI = confidence interval, M-H = Mantel-Haenszel).

Fourteen trials, including 901 subjects, reported the total efficacy rate. As shown in Figure 3, a statistically significant difference was found (*P* < .00001), and a fixed-effects model was used because of mild heterogeneity (*I^2^* = 24%). These results demonstrate a higher efficacy rate in the CHM group.

#### 3.4.2. Cure rate

**Figure 4.**
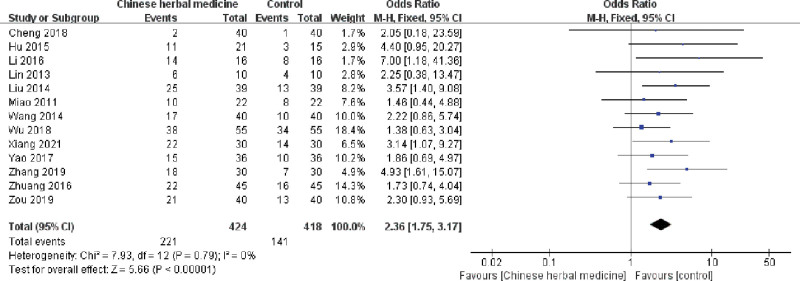
Forest plot showing the effect of Chinese herbal medicine vs controls on the cure rate in the treatment of postcholecystectomy diarrhea (CI = confidence interval, M-H = Mantel-Haenszel).

Thirteen trials including 842 participants reported cure rates. As shown in Figure 4, a statistically significant difference was found (*P* < .00001), and a fixed-effects model was used (*I^2^* = 0%). The results showed a higher cure rate in the CHM group.

#### 3.4.3. T-lymphocyte subset determination

**Figure 5.**
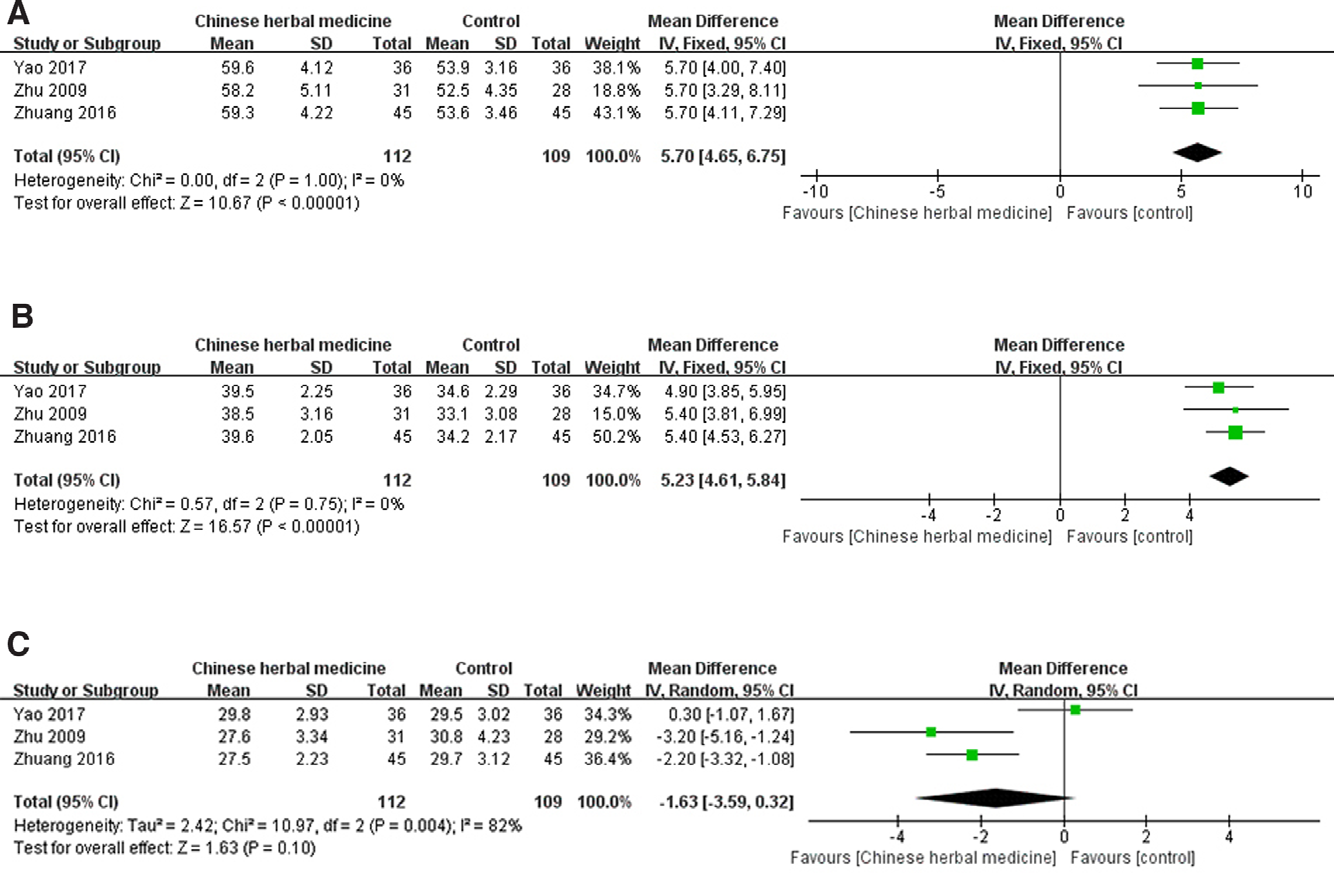
Forest plot showing the effect of Chinese herbal medicine vs controls on the detection of T-lymphocyte subsets including CD_3_^+^ (A), CD_4_^+^ (B) and CD_8_^+^ (C) in the treatment of postcholecystectomy diarrhea (CI = confidence interval, IV = inverse variance).

Three trials including 221 subjects reported on T-lymphocyte subset determination. As shown in Figure 5, statistically significant differences were found in the CD_3_^+^ (*P* < .00001, Fig. 5A) and CD_4_^+^ (*P* < .00001, Fig. 5B) levels, without heterogeneity (*I^2^* = 0%). However, there was no statistically significant difference in the number of CD_8_^+^ cells (*P* = .010, Fig. 5C), with severe heterogeneity (*I^2^* = 82%).

#### 3.4.4. Scores of main symptoms

**Figure 6.**
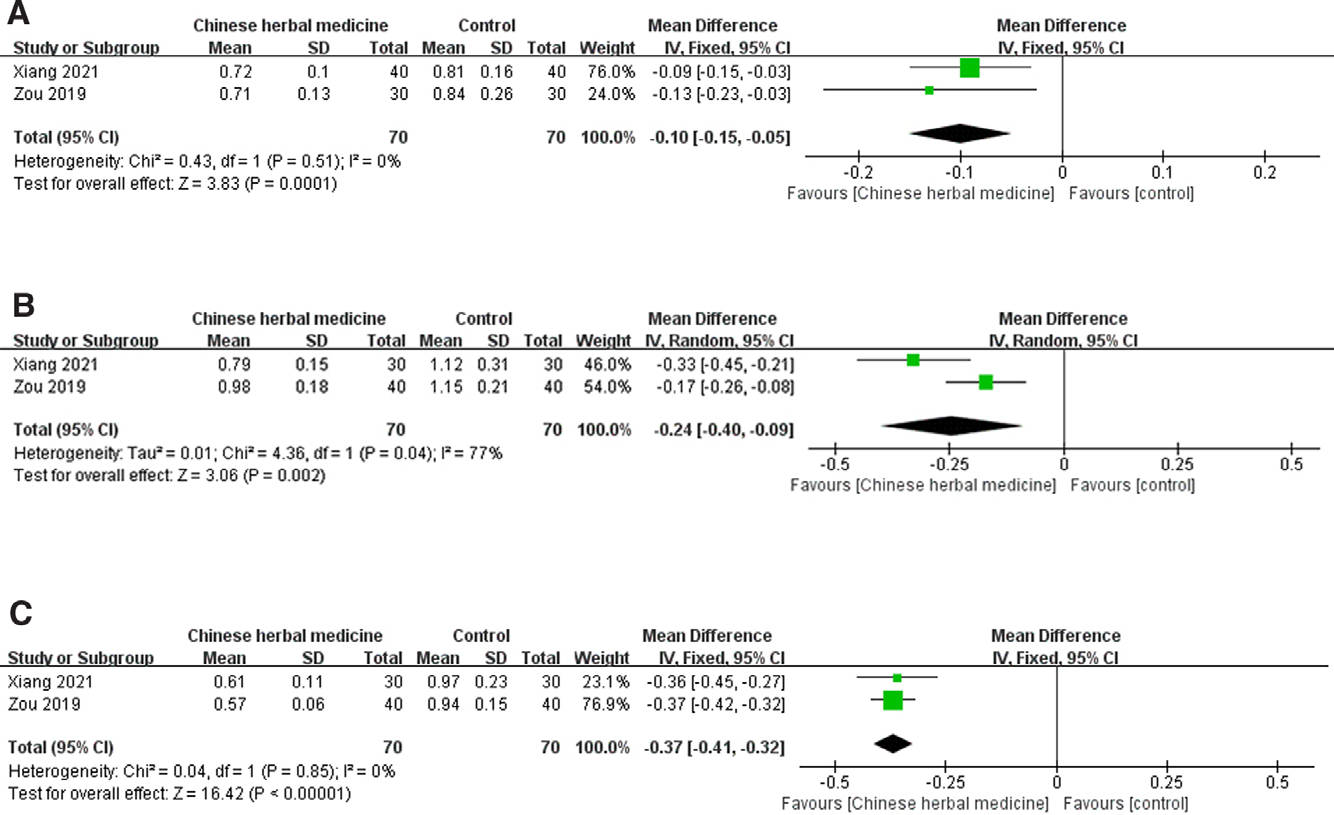
Forest plot showing the effect of Chinese herbal medicine vs controls on the scores of main symptoms including stool nature (A), defecation frequency (B), abdominal pain and bloating (C) in the treatment of postcholecystectomy diarrhea (CI = confidence interval, IV = inverse variance).

Two trials, including 140 participants, reported scores for the main symptoms. As shown in Figure 6, significant differences were found in the nature of stool (*P* < .00001, Fig. 6A), defecation frequency (*P* = .002, Fig. 6B), abdominal pain, and bloating (*P* < .00001, Fig. 6C). A random-effects model was used only for defection frequency owing to severe heterogeneity (*I^2^* = 77%), whereas a fixed-effects model was used in the remaining 2 terms without heterogeneity (*I^2^* = 0%).

### 3.5. Sensitivity analysis

Sensitivity analysis was conducted to evaluate the effect of individual studies on the overall outcome by sequentially removing the studies. Regarding CD_8_^+^, there was a substantial change in the results and heterogeneity when the study by Yao was removed (*P* < .00001, *I^2^* = 0%).

### 3.6. Publication bias

**Figure 7.**
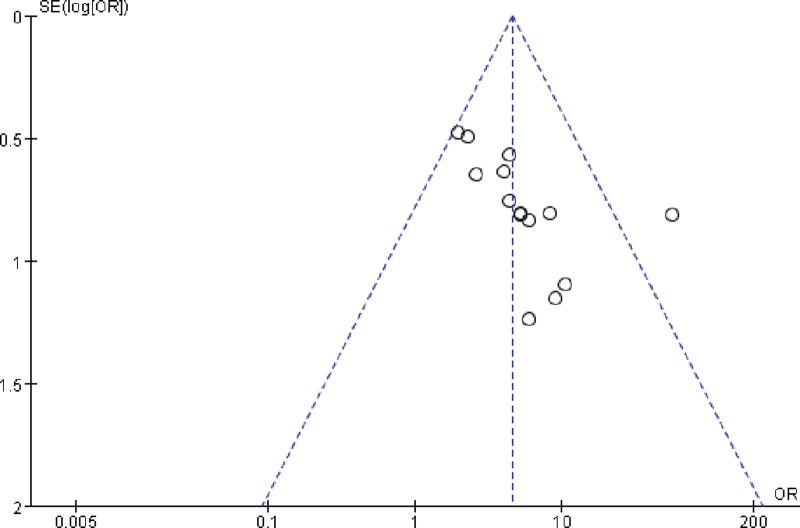
Funnel plot for detecting publication bias for studies comparing the effect of Chinese herbal medicine vs controls on the total effective rate in the treatment of postcholecystectomy diarrhea.

The total effective rate was the common outcome index of the 14 included RCTs and was also the main indicator. Therefore, an outcome index was used to create a funnel plot for detecting publication bias (Fig. 7). Visual inspection of the funnel plots revealed symmetry, suggesting that there was no publication bias.

## 4. Discussion

Diarrhea is one of the most frequently reported postoperative complications of cholecystectomy.^[34]^ Although multiple possible avenues have been proposed, the multifactorial mechanisms underlying the development of PCD remain unclear. A growing number of studies have suggested that hydrophobic bile acid concentrations, increased enterohepatic cycles, lack of gallbladder storage, malabsorption of bile acids, and changes in the gut microbiota may play an important role.^[35–37]^ Nevertheless, Laura et al^[11]^ proposed no significant relationship between the date of cholecystectomy and the onset of bile acid diarrhea. Therefore, current treatments mainly focus on symptom relief.^[17]^ Considering its disabling nature and the lack of effective treatment for PCD, CHM has been widely used as a complementary and alternative therapy based on the pattern of symptoms. The variety of CHM is rich, with the advantages of being well-tolerated, simple to produce, and economical.^[38]^ Here, we conducted a meta-analysis of RCTs on the efficacy and safety of CHM for the treatment of PCD.

Our primary findings indicate that CHM has a higher total effectiveness and cure rate as a monotherapy for PCD. Two trials reported the scores for the main symptoms. Statistically significant differences were observed in the nature of stool, defecation frequency, abdominal pain, and bloating. The main PCD syndrome types in TCM are spleen deficiency and liver stagnation. Generally, the liver and gallbladder are perfectly sympathetic to each other and regulate the qi flow of the entire body. The spleen regulates the movement and transformation of food and water. In addition, the transport function of the spleen relies on the normal drainage function of the liver. After cholecystectomy, the gallbladder loses its ability to function, which leads to liver qi stagnation, transverse invasion into the spleen, and weakness of the spleen transport function, thus causing diarrhea.

Among the included studies, 6 used Tongxie Yaofang, a representative CHM, for diarrhea with pain caused by spleen deficiency and a liver stagnation pattern. Tongxie Yaofang strengthens the spleen, eliminates dampness, soothes the liver, regulates qi, and relieves diarrhea.^[39]^ Several reports have confirmed its effectiveness and few adverse reactions in the treatment of gastrointestinal dysfunction, including diarrhea-predominant irritable bowel syndrome (IBS-D)^[40]^ and ulcerative colitis.^[41]^ Tongxie Yaofang may improve IBS-D by inhibiting apoptosis and inflammatory responses, regulating intestinal flora, relieving visceral hypersensitivity, and enhancing the intestinal mucosal barrier.^[5,42]^ Four studies used Shenling Baizhu Powder, a classical and widely used prescription for managing spleen deficiency diarrhea. Splenic deficiency syndrome is closely related to immune system dysfunction, and CHM may treat splenic deficiency-induced diarrhea by adjusting the absorption function of the intestinal mucosa, improving the inflammatory response, and regulating immunity.^[43]^

In addition, CHM may reduce CD_3_^+^ and CD_4_^+^ T lymphocyte subsets more effectively. Increasing evidence suggests that the immune system plays a vital role in diarrheal pathogenesis.^[38]^ T-lymphocyte subsets are important indicators of cellular immune function. CD_3_^+^ is an indicator of the overall immune status of cells and the total number of T lymphocytes. Based on their phenotypes, T lymphocytes were divided into CD_4_^+^ and CD_8_^+^ subgroups. CD_4_^+^ cells are mainly helper T lymphocytes, whereas CD_8_^+^ cells are mainly inhibitory and cytotoxic T lymphocytes, which dynamically regulate immunity. Patients with IBS may have reduced CD_3_^+^ and CD_4_^+^ counts, which leads to immune function disorders, the release of injurious immune factors, cell damage, persistent inflammatory reactions, and consequent dysfunction of sensory, secretory, and motor functions of the gut.^[44]^ Therefore, we speculate that CHM has the desired holistic and bidirectional immune regulatory functions in PCD. However, the specific mechanism of CHM in PCD remains unclear, and further studies are warranted.

### 4.1. Limitations

To the best of our knowledge, this is the first meta-analysis to evaluate the efficacy of CHM in the treatment of PCD. However, this study has some limitations. First, most studies lacked details on random sequence generation, allocation concealment, blinding of participants and personnel, and blinding of outcome assessment, which may have contributed to the low general level of evidence. Second, considering the individualized prescription characteristics of CHM, there were various CHM prescriptions in the present study. In addition, prescriptions may be modified based on changes in symptoms. These findings are not conducive to the methodological quality of RCTs.^[17]^ Third, the follow-up and treatment times were too heterogeneous to be standardized. Finally, we used outcome measurements that are not commonly used internationally. There is no standardized definition of PCD. However, it is noteworthy that most studies referred to the criteria compiled by TCM experts in China, and we still expected future studies to apply the internationally validated questionnaires for reproducibility. Overall, larger, high-quality RCTs are required, and the study of the mechanisms of CHM and PCD could promote clinical progress.

## 5. Conclusion

Oral CHM is well tolerated, simple, and economical with a desired curative effect, which is worthy of clinical promotion. The results of the present meta-analysis revealed that CHM had a more favorable effect on PCD. No adverse events were observed. However, considering the heterogeneity and generally low quality of the included RCTs, further research is required to draw definitive conclusions and establish standardized treatment protocols.

## Author contributions

**Conceptualization:** Yan Sun, Quanda Liu, Juefei Mo.

**Data curation:** Yan Sun, Yong Zhang.

**Formal analysis:** Yan Sun, Yong Zhang, Zheng Wang.

**Methodology:** Yan Sun, Quanda Liu.

**Validation:** Zheng Wang.

**Writing – original draft:** Yan Sun, Yong Zhang, Quanda Liu.

**Writing – review & editing:** Juefei Mo.
